# *In situ* surface manipulation Mn-based Prussian blue analogues with enhanced redox chemistry and ion diffusion toward high-energy-density aqueous sodium-ion batteries

**DOI:** 10.1039/d5sc07659e

**Published:** 2025-11-18

**Authors:** Hao Fu, Xianpeng Wang, Jun Yang, Zhiqiang Wu, He Ren, Jianeng Ji, Minjie Shi, Edison Huixiang Ang

**Affiliations:** a School of Materials Science and Engineering, Jiangsu University of Science and Technology Jiangsu 212003 P. R. China iamjyang@just.edu.cn; b Macao Institute of Materials Science and Engineering, Macau University of Science and Technology Taipa Macau SAR 999078 China; c Natural Sciences and Science Education, National Institute of Education, Nanyang Technological University Singapore 637616 Singapore edison.ang@nie.edu.sg

## Abstract

Manganese-based Prussian blue analogues (Mn-PBA) are promising cathode materials for aqueous sodium-ion batteries (ASIBs) owing to their open framework that facilitates efficient sodium ion insertion/extraction. However, their practical deployment is hindered by structural collapse arising from high-spin Mn^3+^ (HS-Mn^3+^) dissolution during cycling, triggered by the Jahn–Teller effect, which severely limits long-term stability. Here, we design an *in situ* chemically regulated Mn@Fe/H-PBA electrode with a hierarchical hollow structure *via* co-precipitation. The hollow architecture provides a large surface area for enhanced active site utilization, while the stabilized hierarchical framework enriched with low-spin Mn^3+^ (LS-Mn^3+^) effectively suppresses structural distortion. Together, these features enable Mn@Fe/H-PBA to deliver a high discharge capacity of 121 mA h g^−1^ at 1 A g^−1^ with excellent cycling durability. *In situ*/*ex situ* characterization combined with density functional theory (DFT) calculations confirm the improved redox activity and mitigated Jahn–Teller distortion. Full cells paired with a polyimide (PI) anode achieve an energy density of 74.32 W h kg^−1^, while pouch cells demonstrate stable cycling over 500 cycles at 1 A g^−1^. This work provides a robust strategy to overcome stability challenges in Mn-PBA cathodes for next-generation ASIBs.

## Abstract 2

Mn-PBA was engineered through *in situ* chemical control with a low spin hierarchical structure, which not only demonstrates superior diffusion kinetics in aqueous sodium-ion batteries but also remarkably enhanced utilization of active sites.

## Graphical abstract

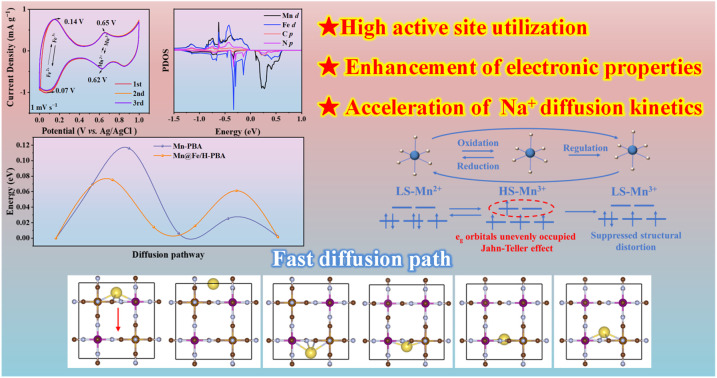

## 1 Introduction

Aqueous sodium-ion batteries (ASIBs) have attracted increasing attention as promising candidates for large-scale energy storage, owing to their intrinsic advantages of non-flammability, low cost, and environmental benignity of aqueous electrolytes.^1–6^ Compared with conventional organic electrolytes, aqueous systems significantly enhance operational safety by mitigating fire and explosion hazards.^7,8^ In addition, sodium-ion batteries (SIBs) provide clear cost benefits over lithium-ion batteries (LIBs), further supporting their scalability for grid-level applications.^9,10^ However, the larger ionic radius of Na^+^ relative to Li^+^ hampers efficient insertion/extraction into host structures, while the narrow electrochemical stability window of aqueous electrolytes constrains the working voltage and limits energy density.^11,12^ Consequently, the rational design and selection of suitable cathode materials is critical to unlocking the practical potential of ASIBs. To date, extensive efforts have focused on cathode systems such as Prussian blue analogues (PBAs), polymers, and transition-metal oxides (*e.g.*, Mn- and V-based).^13,14^

PBAs are a class of compounds obtained by partially substituting metal ions in Prussian blue (PB).^15^ Their open framework facilitates ion insertion/extraction, enables efficient Na^+^ diffusion, and improves coulombic efficiency.^16^ In aqueous electrolytes, PBAs show excellent electrochemical performance along with advantages of simple synthesis, low cost, and environmental friendliness.^13,17^ Manganese-based PBA (Mn-PBA) is particularly attractive due to its high theoretical capacity, low cost, and dual redox couples (Mn^3+^/Mn^2+^ and Fe^3+^/Fe^2+^), which enhance Na^+^ storage and energy density. However, conventional co-precipitation synthesis leads to crystal water vacancies, Mn dissolution (Jahn–Teller effect), sluggish ion diffusion, and poor utilization of redox sites,^18,19^ limiting practical application. To overcome these issues, strategies such as metal co-doping have been developed, which improve conductivity, structural stability, and electrochemical reversibility.^20,21^

Herein, we report an *in situ* chemical regulation strategy coupled with a hydrothermal co-precipitation approach to construct Mn@Fe/H-PBA with a hollow core–shell architecture, comprising manganese-based cores and iron-based shells. This tailored structure delivers three major advantages. First, the low-spin Mn^3+^ configuration effectively suppresses Jahn–Teller distortion, enabling long-term cycling stability over 3000 cycles at high current densities, while expanding the surface area by a factor of 4.36 compared with pristine Mn-PBA. Second, the synergistic interplay between *in situ* regulation and FeHCF incorporation activates dual redox couples (Mn^3+^/Mn^2+^ and Fe^3+^/Fe^2+^), thereby enhancing active-site utilization and significantly improving capacity. Third, accelerated Na^+^ diffusion kinetics are achieved, as confirmed by DFT calculations and *ex situ* EIS analyses, which reveal a lower diffusion barrier and improved charge transport. As a result, Mn@Fe/H-PBA delivers a high specific capacity of 121 mA h g^−1^ at 1 A g^−1^, greatly outperforming Mn-PBA (20 mA h g^−1^) and Mn@Fe-PBA (29 mA h g^−1^). The reaction mechanism and ion-transport behavior were further elucidated through combined DFT simulations and *in situ*/*ex situ* characterizations. When paired with a polyimide anode in a full-cell configuration, Mn@Fe/H-PBA demonstrates outstanding practicality, underscoring its promise as a next-generation cathode material for aqueous sodium-ion batteries.

## 2 Experimental section/methods

### 2.1 Material preparations

#### 2.1.1. Preparation of Mn@Fe/H-PBA

Mn@Fe/H-PBA was synthesized *via* a medium-temperature water bath method. Specifically, 0.5 g of the Mn-PBA precursor was dissolved in 100 mL of deionized water under vigorous stirring. The solution was acidified with 5 mL of 1 M HNO_3_ and subsequently ultrasonicated for 20 min to ensure complete dissolution. After homogenization by magnetic stirring for 5 min, 1 mmol of potassium ferrocyanide (K_4_[Fe(CN)_6_]·3H_2_O) was gradually introduced into the reaction system. The mixture was then hydrothermally treated in a temperature-controlled water bath at 90 °C for 4 h under continuous agitation. The resulting product was recovered by centrifugation at 8000 rpm for 5 min, washed three times with deionized water using redispersion–centrifugation cycles, and finally vacuum-dried at 60 °C for 12 h to obtain the Mn@Fe/H-PBA powder.

#### 2.1.2. Electrochemical measurements

The electrode slurry was prepared by homogenously mixing the active material (Mn@Fe/H-PBA), conductive carbon black, and polyvinylidene fluoride (PVDF) binder in a 6 : 3 : 1 mass ratio. *N*-Methyl-2-pyrrolidone (NMP) was added dropwise to the mixture in an agate mortar while grinding continuously to achieve a uniform dispersion. The resulting slurry was coated onto circular carbon paper substrates (10 mm diameter) using a doctor blade, maintaining an active material loading of ∼1.0 mg cm^−2^. The coated electrodes were vacuum-dried at 60 °C for 12 h to remove residual solvent. Electrochemical evaluation was conducted in a three-electrode system, with carbon rods as the counter electrode, Ag/AgCl as the reference electrode, and 1 M NaCl aqueous solution as the electrolyte. Comprehensive electrochemical analyses, including electrochemical impedance spectroscopy (EIS), galvanostatic charge–discharge (GCD), and cyclic voltammetry (CV), were performed to assess electrode performance.

#### 2.1.3. Computational details

All density functional theory (DFT) calculations for the Mn@Fe/H-PBA and Mn-PBA systems were performed using the Vienna *ab initio* simulation package (VASP).^22^ The projected augmented wave (PAW) method^23^ was employed to describe electron–ion interactions, and the exchange–correlation potential was treated using the Perdew–Burke–Ernzerhof (PBE) functional within the generalized gradient approximation (GGA).^24^ The D3 van der Waals (vdW) correction was applied to account for dispersion interactions.^25^ A plane-wave cutoff energy of 500 eV was adopted, and the energy and force convergence criteria were set to 10^−4^ eV and 0.03 eV Å^−1^, respectively. Transition states for the Mn@Fe/H-PBA and Mn-PBA electrodes were identified using the climbing image nudged elastic band (CI-NEB) method.^26^

## 3 Result and discussion

**Fig. 1:**
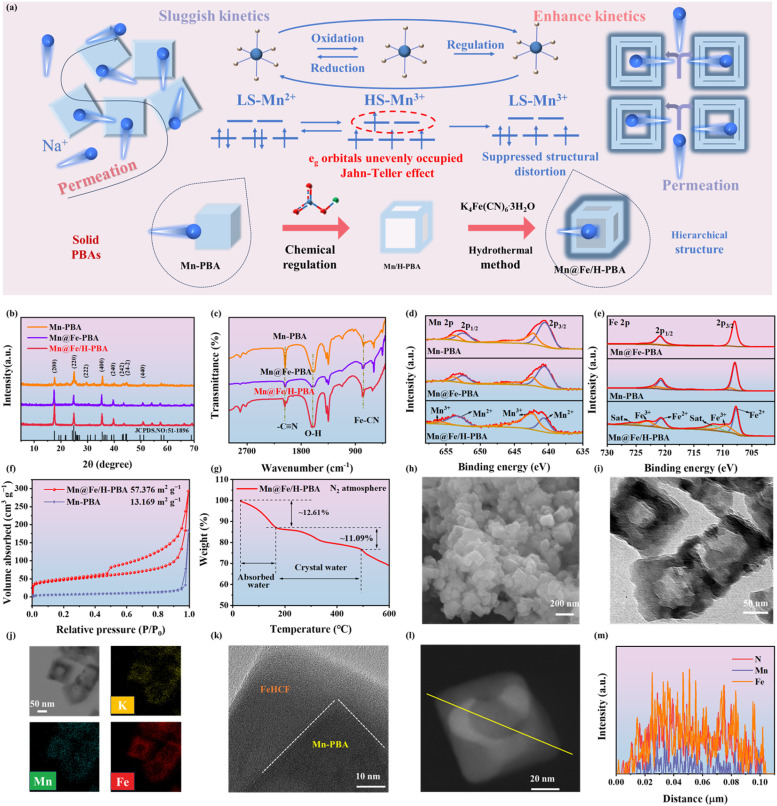
Structural characterization of Mn@Fe/H-PBA. (a) Schematic illustration of the preparation process of Mn@Fe-PBA samples *via* chemical regulation and hydrothermal treatment. (b) XRD patterns, (c) FT-IR spectra, XPS spectra of Mn@Fe/H-PBA of core-levels (d) Mn 2p and (e) Fe 2p, (f) N_2_ adsorption–desorption isotherms, (g) TGA curve, (h) SEM image, (i) TEM image along with the corresponding, (j) elemental mapping images. (k) HRTEM image. (l) HAADF-STEM image. (m) Laser EDS results of the yellow lines in (l).

Fig. 1a illustrates the synthesis of Mn@Fe/H-PBA. Conventional manganese-based Prussian blue analogues (Mn-PBAs) often suffer from structural degradation due to Jahn–Teller distortion and progressive capacity decay during sodium-ion (de)intercalation. To overcome these limitations, an *in situ* modulation strategy was employed, involving the epitaxial growth of iron hexacyanoferrate (FeHCF). This process induced a high-spin to low-spin transition in Mn^3+^ ions, yielding a stabilized Mn@Fe/H-PBA composite that effectively suppressed Jahn–Teller distortion and mitigated rapid capacity decay during cycling. The engineered Mn@Fe/H-PBA exhibited a hollow hierarchical structure, which provided dual functionality: it significantly increased the specific surface area, exposing abundant electroactive sites and enhancing reaction kinetics, while maintaining structural integrity during repeated charge/discharge cycles. X-ray diffraction (XRD) analysis (Fig. 1b) confirmed the formation of a monoclinic K_*x*_MnFe(CN)_6_·*y*H_2_O phase (JCPDS No. 51-1896), with enhanced peak intensities relative to Mn-PBA indicating improved crystallinity.^27^ Raman spectroscopy revealed Fe–CN stretching vibrations at 2000–2200 cm^−1^ (Fig. S1),^28^ while FT–IR analysis (Fig. 1c) showed peaks at 596 cm^−1^ and 2071 cm^−1^ corresponding to Fe–CN and CN stretching, respectively.^29^ Notably, a distinct LS-Mn^3+^–N vibration was observed at 2043 cm^−1^ (Fig. S2), contrasting with the LS-Mn^2+^–N vibration near 2030 cm^−1^ in Mn-PBA, confirming the successful spin-state modulation.^30,31^ The peak at 1632 cm^−1^ was attributed to O–H vibrations of coordinated water, consistent with thermogravimetric analysis (Fig. S3a and b). X-ray photoelectron spectroscopy (XPS) further validated the chemical composition and valency states. Mn 2p spectra showed Mn^2+^ peaks at 640.68 eV (2p_3/2_) and 653.38 eV (2p_1/2_), and Mn^3+^ peaks at 642.38 eV and 656.08 eV. Fe 2p spectra revealed Fe^2+^ at 707.68 eV and 720.68 eV and Fe^3+^ at 709.58 eV and 723.08 eV, with satellite peaks at 711.78 eV and 725.78 eV. After modification, a shift in Fe satellite peaks was observed, and comparative analysis showed higher Mn^3+^ and Fe^3+^ content in Mn@Fe/H-PBA (Fig. S5), confirming successful LS-Mn^3+^ incorporation. Increased peak areas in N 1s XPS spectra further indicated enhanced Mn–N bonding (Fig. S6 and S7). The N 1s XPS spectra of Mn@Fe/H-PBA revealed a pronounced increase in the Mn–N peak area, confirming the successful incorporation of LS-Mn^3+^. BET analysis (Fig. 1f and S7) showed a specific surface area of 57.38 m^2^ g^−1^, approximately 4.36 times higher than that of Mn-PBA (13.17 m^2^ g^−1^), indicating enhanced exposure of electroactive sites and improved reaction kinetics. Thermogravimetric analysis (Fig. 1g) revealed higher bound water content in Mn@Fe/H-PBA, consistent with its hollow architecture and corroborated by FT-IR spectra (Fig. S3). Morphological characterization by SEM and TEM (Fig. 1h–i and S9–S11) confirmed a uniform cubic structure and well-defined hollow core–shell architecture. SEM–EDS mapping (Fig. S10) demonstrated homogeneous distribution of Mn and Fe, while TEM elemental mapping (Fig. 1j) revealed Fe enrichment in the shell and Mn concentration in the core, validating the spatial segregation. The HRTEM image (Fig. 1k) clearly reveals a heterostructure. The corresponding EDS line scan along one of the diagonals shows that Fe is mainly distributed in the outer region, whereas Mn is concentrated in the inner region (Fig. 1l and m). Collectively, these results confirm that the rationally designed Mn@Fe/H-PBA possesses a well-ordered, hollow hierarchical structure that facilitates efficient Na^+^ intercalation/deintercalation.

**Fig. 2:**
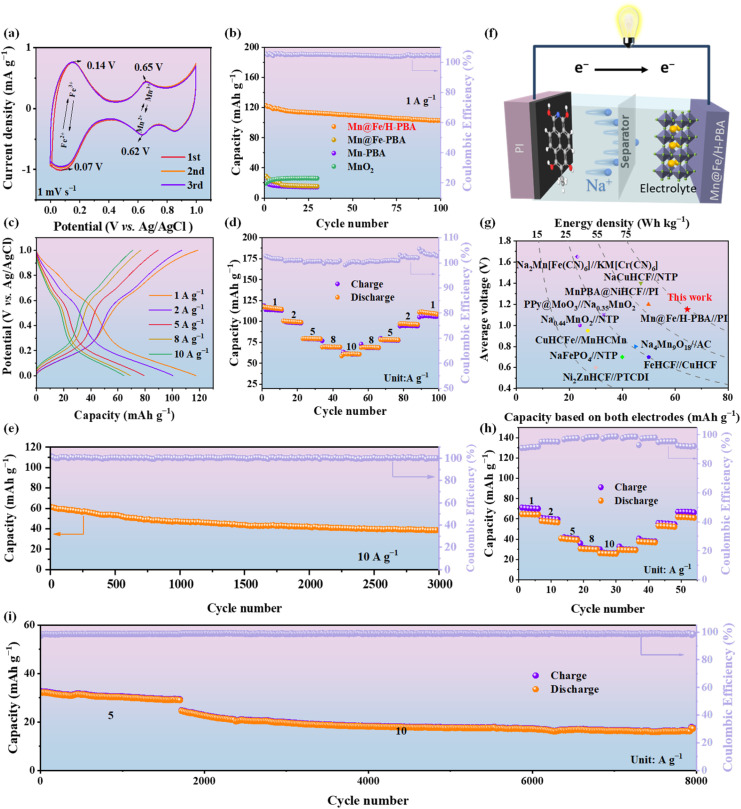
Electrochemical characterization of Mn@Fe/H-PBA as cathode: (a) CV curves for the first three cycles recorded at 1 mV s^−1^; (b) cycling performance comparison of Mn@Fe/H-PBA with Mn@Fe-PBA, Mn-PBA, and MnO_2_; (c and d) rate performance of Mn@Fe/H-PBA at various current densities; (e) long-term cycling stability of Mn@Fe/H-PBA at 10 A g^−1^. Electrochemical performance and full-cell application of Mn@Fe/H-PBA//PI//Na: (f) schematic illustration of the full cell; (g) average voltage, specific capacity, and energy density based on total electrode mass; (h) rate performance; and (i) long-term cycling performance at 5 and 10 A g^−1^.

The electrochemical properties of the materials were systematically evaluated using cyclic voltammetry (CV) and galvanostatic charge–discharge (GCD) in a three-electrode configuration. As shown in Fig. 2a, CV of Mn@Fe/H-PBA at a scan rate of 1 mV s^−1^ exhibited two well-defined redox couples at *E*_1_ = 0.07/0.14 V and *E*_2_ = 0.62/0.65 V *vs.* Ag/AgCl, corresponding to the Fe^2+^/Fe^3+^ and Mn^2+^/Mn^3+^ redox processes. This dual redox behaviour confirms the effective implementation of a dual-center electron transfer mechanism, allowing full utilization of both manganese-based coordination sites and iron-centered redox-active units.^31,32^ The near-overlapping CV curves indicate excellent electrochemical stability. Comparison with Mn-PBA and Fe-PBA (Fig. S12) highlights that the synergistic effect of the dual active sites underpins the high specific capacity of Mn@Fe/H-PBA. GCD measurements (Fig. 2b) further demonstrated that Mn@Fe/H-PBA delivers an ultra-high initial discharge capacity of 121 mA h g^−1^ with excellent coulombic efficiency, outperforming Mn@Fe-PBA, Mn-PBA, and MnO_2_ electrodes (Fig. S13 and Table S1). These results underscore the advantages of the hollow core–shell architecture and dual redox centers in enhancing electrochemical performance.^33–38^Fig. 2c presents the GCD curves of Mn@Fe/H-PBA at various current densities, consistent with the CV results. Two well-defined charge–discharge plateaus were observed in the first three cycles at 1 A g^−1^, and the near-overlapping curves indicate excellent electrochemical stability (Fig. S14). As shown in Fig. 2d, Mn@Fe/H-PBA exhibited remarkable rate capability and reversibility, delivering discharge capacities of 117.39, 100.57, 79.31, 69.56, and 60.56 mA h g^−1^ at current densities of 1, 2, 5, 8, and 10 A g^−1^, respectively. When the current density was returned to 1 A g^−1^, the discharge capacity recovered to 111.93 mA h g^−1^, confirming good reversibility. Long-term cycling at 10 A g^−1^ for 3000 cycles (Fig. 2e) demonstrates excellent stability, maintaining a capacity retention of approximately 65.6%. SEM images further reveal negligible structural changes before and after cycling (Fig. S15). To further evaluate practical applicability, a full cell was constructed using Mn@Fe/H-PBA as the cathode and commercial polyimide (PI)as the anode (Fig. 2f). The full cell exhibited a wide voltage window of 2.1 V and four pairs of redox peaks at scan rates of 1–8 mV s^−1^ (Fig. S16). Fig. 2g compares the average voltage and energy density of this system with previously reported materials, revealing a high average voltage of 1.15 V and an energy density of 74.32 W h kg^−1^ (Table S2), highlighting the excellent practical potential of Mn@Fe/H-PBA for aqueous sodium-ion batteries.^7,39–47^

**Fig. 3:**
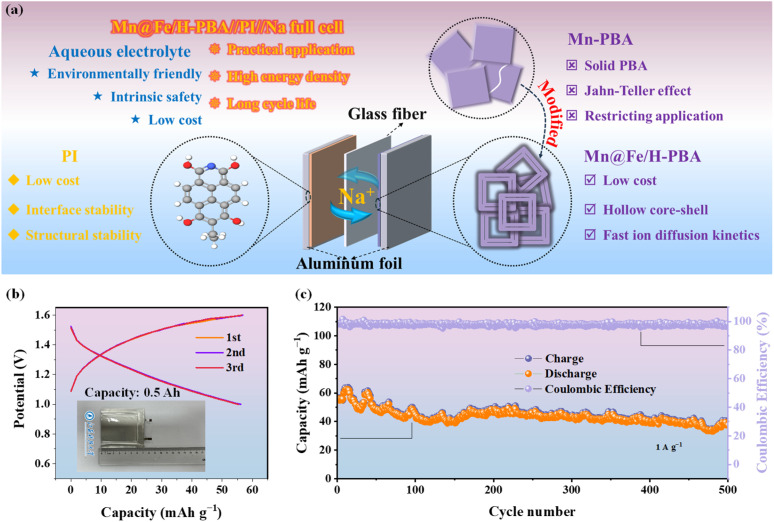
Electrochemical performances of the Mn@Fe/H-PBA//PI//Na pouch cell. (a) Exploded-view schematic illustrating the pouch cell design and key advantages. (b) GCD profiles for the first three cycles at 1 A g^−1^ (inset: photograph of the assembled pouch cell). (c) Long-term cycling performance over 500 cycles, demonstrating excellent stability and durability.

The electrochemical behavior of PI is depicted in Fig. S17a and S18. As shown in Fig. S17c, the CV curves of the positive electrode, negative electrode, and full cell at 1 mV s^−1^ display well-defined redox features. The rate capability of the full cell is presented in Fig. 2h, exhibiting excellent performance at current densities of 5 and 10 A g^−1^. Long-term cycling at these rates (Fig. 2i and S19) further confirms superior stability, sustaining 8000 cycles with negligible performance loss (capacity retention of 70% after 6000 cycles at 10 A g^−1^), thereby highlighting the commercial relevance of Mn@Fe/H-PBA. To further validate the practical feasibility of the Mn@Fe/H-PBA//PI//Na configuration, electrochemical tests were performed in an assembled pouch cell using an aluminum foil current collector, glass fiber separator, and 1 M NaCl electrolyte. As illustrated in Fig. 3a, the system demonstrates considerable promise for practical deployment owing to the environmental benignity, intrinsic safety, and low cost of its components. Representative GCD curves for the initial three cycles at 1 A g^−1^ (Fig. 3b, inset) exhibit nearly perfect overlap, evidencing excellent electrochemical reversibility. Extended cycling at 1 A g^−1^ over 500 cycles further reveals outstanding durability, with an ultralow average capacity fading rate of only 0.06% per cycle. Such exceptional stability underscores the strong potential of this system for practical energy storage applications.

**Fig. 4:**
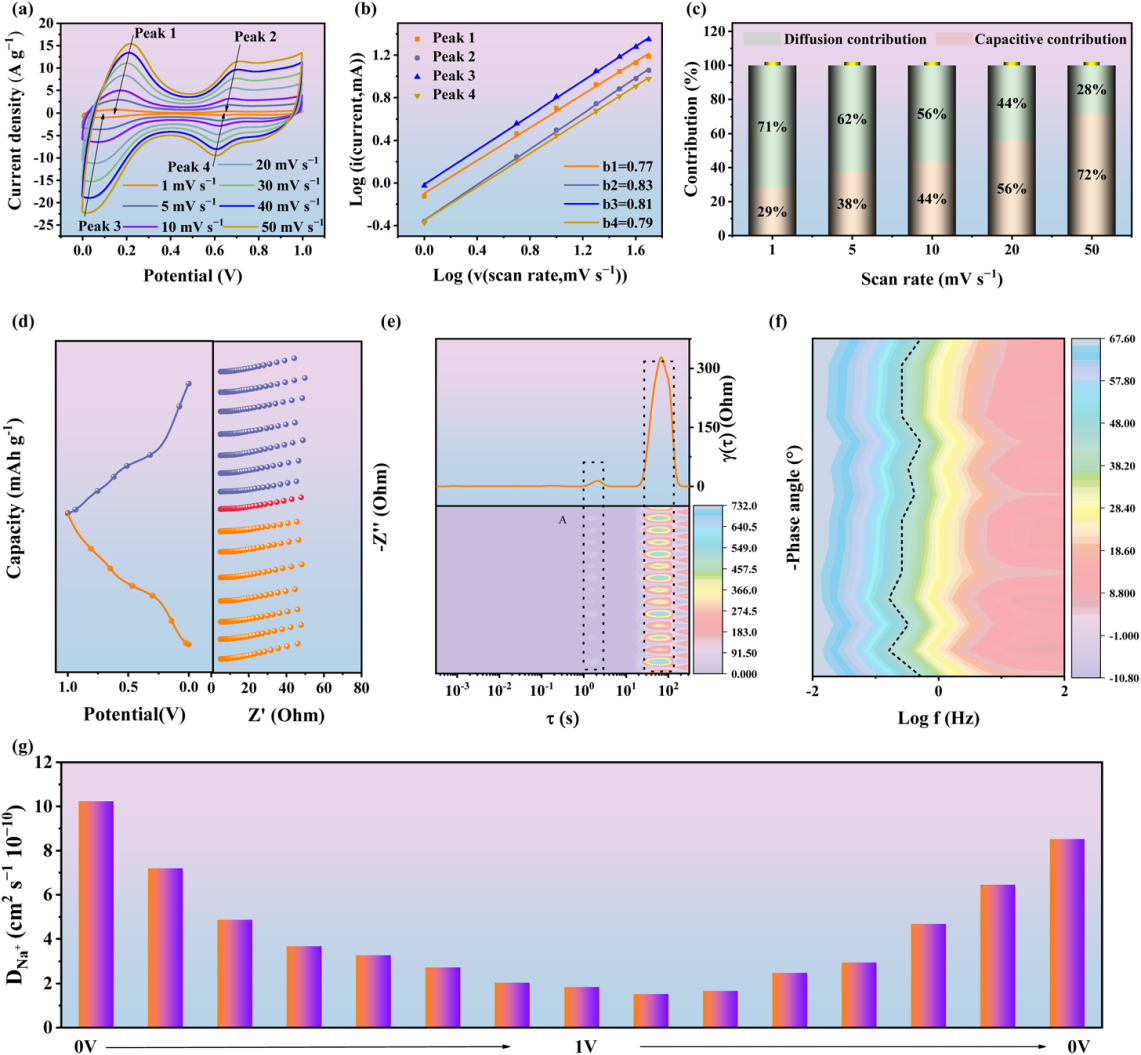
Electrochemical characterization of Mn@Fe/H-PBA. (a) CV curves recorded at multiple scan rates. (b) Plots of ln(*i*) *versus* ln(*v*) for the corresponding redox peak currents. (c) Quantitative analysis of capacitive contribution at different scan rates. (d) EIS spectra measured at various states of charge. (e) Distribution of relaxation times (DRT) derived from the EIS data. (f) Bode plots at different applied potentials, illustrating frequency-dependent electrochemical behavior, and (g) Na^+^ diffusion.

To further elucidate the sodium-storage kinetics of Mn@Fe/H-PBA, CV measurements were conducted at scan rates ranging from 1 to 50 mV s^−1^ (Fig. 4a). Two distinct pairs of redox peaks were observed, corresponding to Fe^2+^/Fe^3+^ and Mn^2+^/Mn^3+^ centers. The peak positions remained nearly unchanged with increasing scan rate, confirming the robust activation of redox-active sites and excellent reversibility of the electrode. The redox kinetics were analyzed using the power–law relationship *i* = *av*^*b*^.^48,49^ The fitted *b* values (0.77–0.83) indicate a mixed charge–storage process governed by both pseudocapacitive and diffusion–controlled contributions (Fig. 4b). Quantitative analysis using *i* = *k*_1_*v* + *k*_2_*v*^0.5^ further revealed that the capacitive contribution progressively increased with scan rate,^50^ from 29% at 1 mV s^−1^ to 72% at 50 mV^−1^ (Fig. 4c and S20a–e). These results demonstrate that the synergistic interplay of diffusion and pseudocapacitance underpins the superior kinetics of Mn@Fe/H-PBA. Electrochemical impedance spectroscopy (EIS) provided additional insight into charge-transfer dynamics (Fig. S21 and Table S3).^51^ Compared with Mn-PBA, Mn@Fe/H-PBA exhibited significantly lower equivalent series resistance and charge-transfer resistance, together with a steeper slope in the low-frequency region, confirming enhanced Na^+^ diffusion and improved conductivity. *Ex situ* EIS at different charge–discharge states (Fig. 4d) showed consistent Nyquist profiles, with equivalent circuit modeling enabling quantitative extraction of resistance parameters. Distribution of relaxation time (DRT) analysis (Fig. 4e, g and Table S4) further revealed dynamic variations in ion transport: the high-frequency relaxation peak initially increased and then decreased, suggesting that Na^+^ diffusion first slowed and then accelerated during cycling. The corresponding Bode plots (Fig. 4f) showed characteristic phase angles near 45° across all voltages,^52^ corroborating rapid charge transfer and fast electrochemical response. These results demonstrate that Mn@Fe/H-PBA delivers ultrafast redox kinetics and exceptional sodium-storage dynamics.

To further elucidate the sodium storage mechanism, Mn@Fe/H-PBA was systematically characterized using *in situ*.

**Fig. 5:**
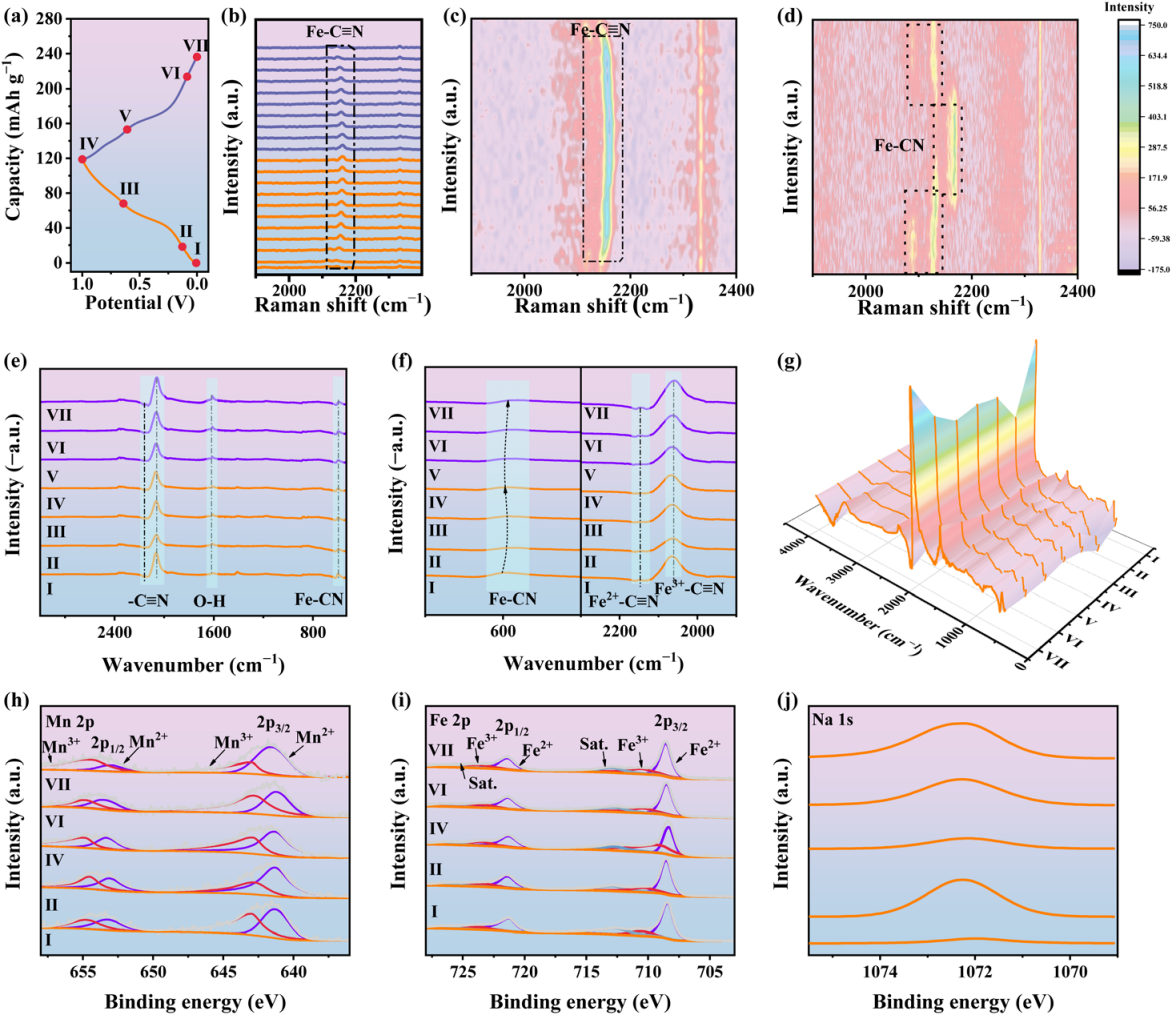
Sodium-ion storage performances of the Mn@Fe/H-PBA and Mn-PBA electrodes. (a) Representative GCD profile of the Mn@Fe/H-PBA electrode. (b and c) *In situ* Raman spectra and corresponding contour plots of the Mn@Fe/H-PBA electrode. (d) *In situ* Raman spectral analysis of the Mn-PBA electrode for comparison. (e–g) *Ex situ* FT–IR spectra highlighting structural evolution of the Mn@Fe/H-PBA electrode. *Ex situ* XPS spectra of (h) Mn 2p, (i) Fe 2p, and (j) Na 1s for the Mn@Fe/H-PBA electrode during charge–discharge cycles.

Raman spectroscopy, *ex situ* FT-IR, and *ex situ* XPS analyses (Fig. 5a–j). The representative GCD profile during charge–discharge is shown in Fig. 5a. *In situ* Raman contour plots (Fig. S22a) reveal distinct variations in the Mn–N vibrational modes within the 350–700 cm^−1^ range. Specifically, the Mn^2+^–N signal weakens, while the Mn^3+^–N vibration intensifies upon charging, consistent with the reversible Mn^2+^/Mn^3+^ redox process; the opposite trend is observed during discharge. In contrast, Mn-PBA exhibits negligible spectral changes (Fig. S22b), confirming that the rationally designed Mn@Fe/H-PBA markedly enhances Mn redox activity. Likewise, Raman tracking of Fe–CN vibrations (Fig. 5b–c and S23) demonstrates reversible Fe^2+^ → Fe^3+^ oxidation upon charging and subsequent reduction during discharge, without any peak disappearance, indicating excellent structural integrity and the absence of phase transitions. Conversely, the *in situ* Raman spectra of Mn-PBA (Fig. 5d and S23), in the 2000–2200 cm^−1^ region reveal a detrimental phase transition that undermines its structural stability.^53,54^ Complementary *in situ* FT-IR measurements (Fig. 5e–g) revealed corresponding reversible shifts in Fe–CN stretching vibrations (∼2300 cm^−1^) and bending modes (∼590 cm^−1^), further confirming the Fe^2+^/Fe^3+^ redox activity of the [Fe(CN)_6_]^4−^/[Fe(CN)_6_]^3−^ moieties as key capacity contributors. Meanwhile, the O–H vibrational mode (∼1630 cm^−1^) remained nearly unchanged, highlighting the robustness of the framework. *Ex situ* XPS analysis corroborated these findings. The Mn 2p spectra (Fig. 5h) showed reversible Mn^2+^/Mn^3+^ transitions, while the Fe 2p spectra (Fig. 5i) confirmed the participation of Fe redox centers, fully consistent with the *in situ* Raman and FT-IR observations. Importantly, Na 1s spectra (Fig. 5j, S24a and b) revealed clear, reversible insertion/extraction of Na^+^ ions during cycling. These results establish that the superior electrochemical performance of Mn@Fe/H-PBA arises from highly reversible and synergistic Mn^2+^/Mn^3+^ and Fe^2+^/Fe^3+^ redox processes, coupled with stable Na^+^ intercalation dynamics within the robust hollow framework.

**Fig. 6:**
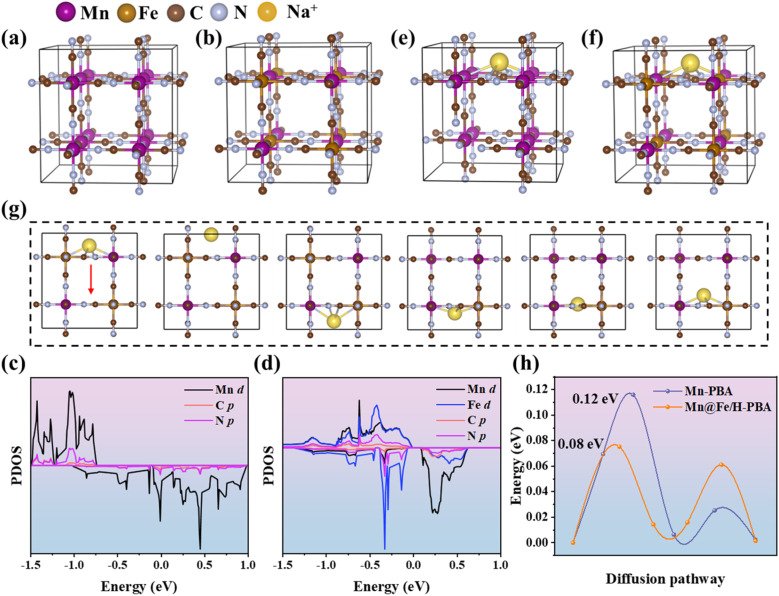
The structural models of (a) Mn-PBA and (b) Mn@Fe/H-PBA. Projected density of states (PDOS) for (c) Mn-PBA and (d) Mn@Fe/H-PBA. Na^+^ adsorption configurations on (e) Mn-PBA and (f) Mn@Fe/H-PBA. (g) Diffusion pathway of Na^+^ in Mn@Fe/H-PBA, and (h) comparison of Na^+^ diffusion energy barriers in Mn-PBA and Mn@Fe/H-PBA.

To elucidate the origin of the outstanding rate performance of Mn@Fe/H-PBA as a cathode for ASIBs, density functional theory (DFT) calculations were carried out. Guided by the experimental results, 50% of the Mn in pristine Mn-PBA was substituted with Fe to construct Mn@Fe/H-PBA, while the coordination environment was precisely regulated to favor the Fe–C≡N–Mn configuration (Fig. 6a and b). The structural stability was first assessed *via* formation energy, where a more negative value denotes enhanced stability. Mn@Fe/H-PBA exhibited a calculated formation energy of −0.11 eV, confirming its excellent thermodynamic stability. The projected density of states (PDOS) was subsequently analyzed to probe the electronic structure (Fig. 6c and d). Compared with Mn-PBA, distinct peaks of Fe d orbitals emerge near the Fermi level in Mn@Fe/H-PBA, demonstrating that Fe incorporation markedly tailors the electronic configuration. The Na adsorption behavior was then evaluated (Fig. 6e and f). In Mn-PBA, Na ions preferentially occupy the hollow site coordinated by four Mn atoms with a favorable adsorption energy of −2.54 eV. In contrast, Mn@Fe/H-PBA stabilizes Na ions at the hollow site coordinated by two Mn and two Fe atoms, maintaining a comparable adsorption energy (−2.53 eV), thereby preserving favorable Na accommodation. To further clarify the ion transport kinetics, Na migration pathways were examined (Fig. 6g, h and S25). High-rate cathodes demand a low diffusion barrier to ensure rapid ion transport. For pristine Mn-PBA, the Na diffusion barrier was calculated to be 0.12 eV, whereas Fe incorporation reduces the barrier to 0.08 eV. This Mn–Fe synergistic effect effectively accelerates Na insertion and extraction, rationalizing the superior rate capability observed experimentally. We also examined whether the presence of K within the framework influences Na migration. The calculated energy barriers are 0.10 eV for Mn-PBA and 0.08 eV for Mn@Fe/H-PBA (Fig. S26), consistent with the values obtained in the absence of K. This indicates that the presence of K does not hinder the rapid migration of Na in Mn@Fe/H-PBA.

## 4 Conclusions

In summary, a hollow hierarchical Mn@Fe/H-PBA was rationally synthesized *via* chemical modulation. The hollow framework afforded a high surface area and abundant accessible sites, thereby enhancing specific capacity, while the low-spin hierarchical configuration effectively mitigated the Jahn–Teller distortion inherent to Mn-PBAs, leading to superior structural stability. As a result, the Mn@Fe/H-PBA cathode exhibited remarkable electrochemical performance in ASIBs, including an ultrahigh reversible capacity of 121 mA h g^−1^ at 1 A g^−1^, outstanding rate capability, and long-term cycling stability. *In situ* and *ex situ* characterizations revealed that the dual redox activity of Fe^2+^/Fe^3+^ and Mn^2+^/Mn^3+^ sites played a critical role in facilitating efficient Na^+^ storage. Moreover, when coupled with PI, a Mn@Fe/H-PBA//PI full cell delivered an energy density of 74.32 W h kg^−1^ with an average potential of 1.15 V, and the assembled 0.5 Ah pouch cell retained stable capacity over 500 cycles. This work highlights a robust strategy for advancing PBAs toward high-performance aqueous energy storage systems.

## Author contributions

J. Yang, H. Fu, and E. H. Ang developed the conceptual framework and designed the experiments. J. Yang and H. Fu were responsible for material fabrication. H. Fu also performed data measurement and analysis, while X. Wang provided DFT calculations. Z. Wu, H. Ren, and J. Ji conducted data investigation. M. Shi handled data analysis. The manuscript was prepared by J. Yang, and E. H. Ang. Funding support was provided by J. Yang and E. H. Ang. H. Fu and X. Wang contributed equally to this work. All authors contributed to discussions and provided feedback on the manuscript.

## Conflicts of interest

There are no conflicts to declare.

## Supplementary Material

SC-017-D5SC07659E-s001

## Data Availability

All relevant data are within the manuscript and the supplementary information (SI). Supplementary information: material preparation method, materials characterizations and electrochemical performance of Mn@Fe/H-PBA and other samples. See DOI: https://doi.org/10.1039/d5sc07659e.
