# Supplementary material for: In situ surface manipulation Mn-based Prussian blue analogues with enhanced redox chemistry and ion diffusion toward high-energy-density aqueous sodium-ion batteries
Source: Chem Sci. 2025 Nov 18;17(2):968–76. doi: 10.1039/d5sc07659e (PMC12626643; doi:10.1039/d5sc07659e)
Supplement: SC-017-D5SC07659E-s001 [file SC-017-D5SC07659E-s001.pdf]

Supplementary Information (SI) for Chemical Science.
This journal is © The Royal Society of Chemistry 2025

In situ surface manipulation Mn−based Prussian blue analogues with enhanced redox chemistry and ion diffusion toward high−energy−density aqueous sodium−ion batteries

Hao Fu[a], Xianpeng Wang[b], Jun Yang[a*], Zhiqiang Wu[a], He Ren[a], Jianeng Ji[a], Minjie Shi[a], Edison Huixiang Ang[c*]

[a]School of Materials Science and Engineering, Jiangsu University of Science and Technology, Zhenjiang, 212003, Jiangsu, P. R. China

[b]Macao Institute of Materials Science and Engineering, Macau University of Science and Technology, Taipa, Macau SAR 999078, China

[c]Natural Sciences and Science Education, National Institute of Education, Nanyang Technological University, Singapore 637616, Singapore

*Corresponding authors.
E-mail address: iamjyang@just.edu.cn (J. Yang); edison.ang@nie.edu.sg (E. H. Ang)

Material preparations

Preparation of Mn-PBA

$K_4Fe(CN)_6 \cdot 3H_2O$ (AR), $FeSO_4 \cdot 7H_2O$ (AR), $MnAc_2 \cdot 4H_2O$ (AR) and EDTA dipotassium salt (AR) were purchased from Shanghai Aladdin and used without further treatment. A total of 1.61 g of EDTA and 4 mmol of manganese acetate were dissolved together in 50 mL of deionized water (denoted as liquid A), while 4 mmol of potassium ferrocyanide was dissolved in 50 mL of deionized water (denoted as liquid B). Liquid A was then slowly added dropwise to liquid B under stirring for 12 min, after which the mixture was left to stand at room temperature for 4 h. The resulting product was collected by centrifugation, washed three times with deionized water, and subsequently dried at 60 °C at room temperature for 12h to obtain Mn-PBA.

Preparation of Mn@Fe/H-PBA

A total of 0.5 g of the previously synthesized Mn-PBA was dissolved in 100 mL of deionized water. After complete dissolution, 5 mL of 1 M HNO_3 was added, and the

mixture was subjected to ultrasonication. The solution was then stirred for 5 min, followed by the addition of 1 mmol of potassium ferrocyanide. The reaction mixture was heated in a 90°C water bath for 4 h, after which the product was collected by centrifugation, washed three times with deionized water, and dried at 60°C for 12 h at room temperature to obtain the Mn@Fe/H-PBA powder. The Mn@Fe-PBA powder was prepared following the same procedure as Mn@Fe/H-PBA, except that the acid treatment step was omitted.

Material characterizations

A Bruker D8 X-ray diffractometer (Cu Kα radiation, λ = 1.5406 Å, 40 kV, 40 mA) was employed to characterize the Mn@Fe-PBA samples via X-ray diffraction (XRD). The diffraction angles were measured from 10 to 70° with a step size of 0.02° and a dwell time of 20 s per step. The morphology of the products was examined using field emission scanning electron microscopy (SEM). The powder samples were dispersed in anhydrous ethanol, ultrasonically treated, and then dropped onto copper foil by capillary action. Prior to imaging, the samples were sputter-coated with gold using an FEI Sirion 200 at an acceleration voltage of 5 kV. Transmission electron microscopy (TEM, JEM-2100F) was also performed for structural analysis. The Mn@Fe/H-PBA samples were analyzed using X-ray photoelectron spectroscopy (XPS) on an ESCALAB250Xi system to determine the valence states of the constituent elements. Chemical bonding was investigated using Fourier-transform infrared spectroscopy (FT-IR) and Raman spectroscopy. FT-IR measurements were conducted with a Thermo Nicolet system, while Raman spectra were recorded using a Renishaw inVia Qontor with a 532 nm laser. Additionally, in situ Raman measurements were carried out using an

electrochemical workstation while performing cyclic voltammetry (CV) tests between 0 and 1 V to monitor real-time changes in the electrode materials.

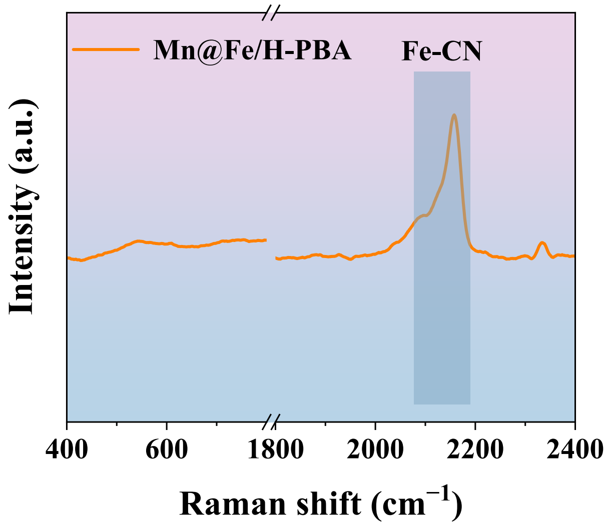

Fig. S1. Raman spectrum of Mn@Fe/H-PBA.

As shown in Fig. S1, the broad peak observed at 400–600 cm^{-1} corresponded to Mn–N stretching vibrations, while the broad peak at 2000–2200 cm^{-1} was attributed to Fe–CN stretching vibrations.

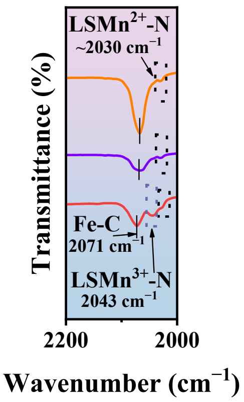

Fig. S2. Magnified view of the FT-IR spectra.

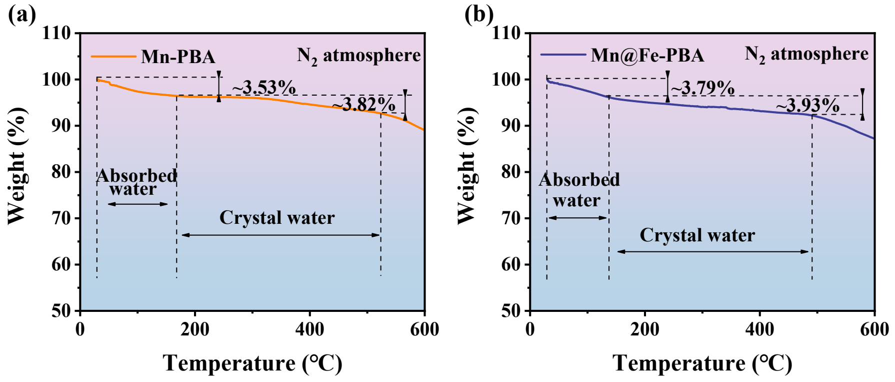

Fig. S3. TG curves of (a) Mn-PBA and (b) Mn@Fe-PBA.

Analysis of the TG curves for the two samples revealed that Mn@Fe/H-PBA exhibited the highest adsorbed water content. This was attributed to the large specific surface area of its hollow structure, consistent with the FT-IR results.

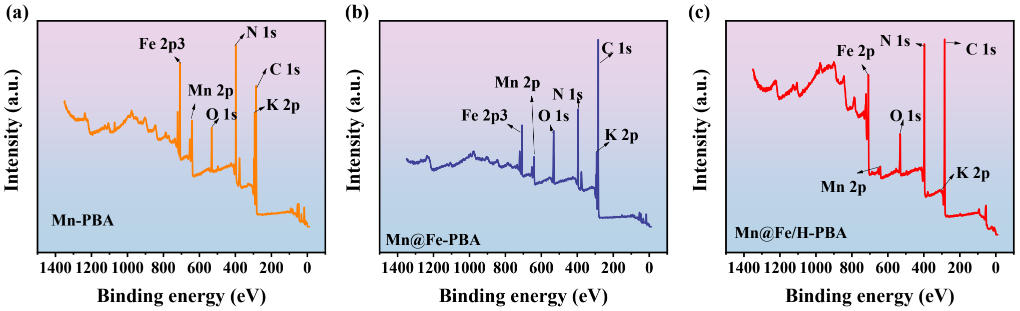

Fig. S4. XPS survey scans of (a) Mn-PBA, (B) Mn@Fe-PBA, and (C) Mn@Fe/H-PBA.

The presence of Mn, Fe, K, C, N, and O elements in the samples was clearly observed in Fig. S4.

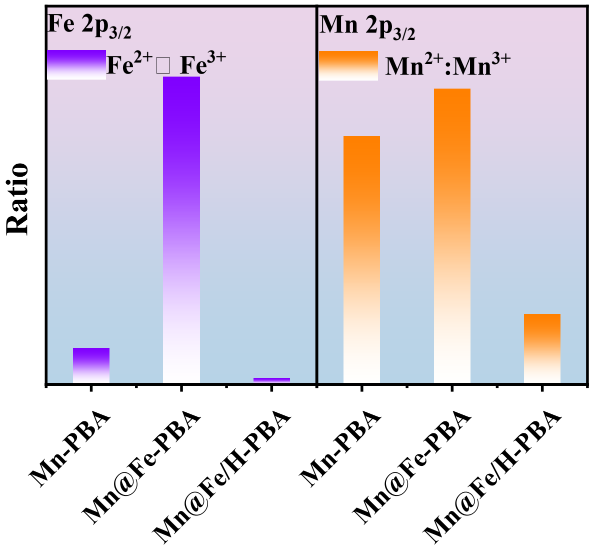

Fig. S5. The proportion of chemical valence states of Mn and Fe in the different powders.

As shown in the figure S5, Fe^{2+} and Mn^{2+} were oxidized to Fe^{3+} and Mn^{3+}, respectively, under the acidic environment. This observation confirmed that the synthesized Mn@Fe/H−PBA achieved a low-spin steady state, supporting the excellent properties of the resulting material.

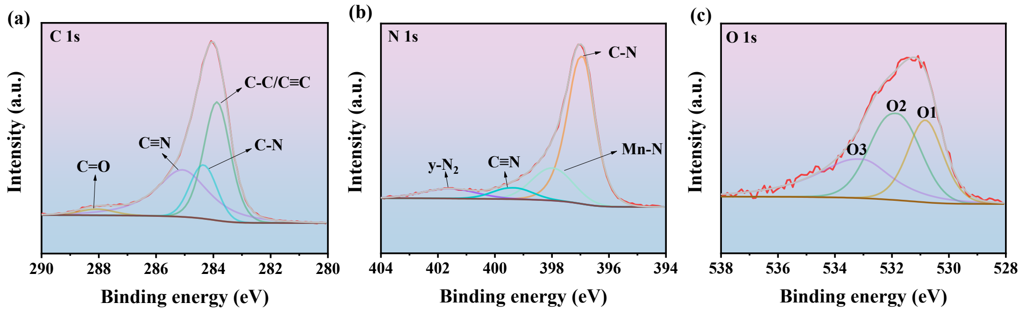

Fig. S6. The XPS spectra of Mn@Fe/H-PBA of core-levels of (a) C 1s, (b) N 1s, and (c) O 1s.

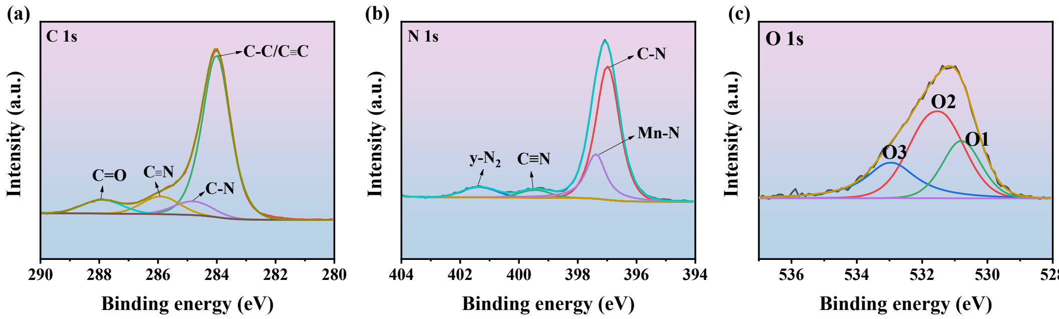

Fig. S7. The XPS spectra of Mn-PBA of core-levels of (a) C 1s, (b) N 1s, and (c) O 1s.

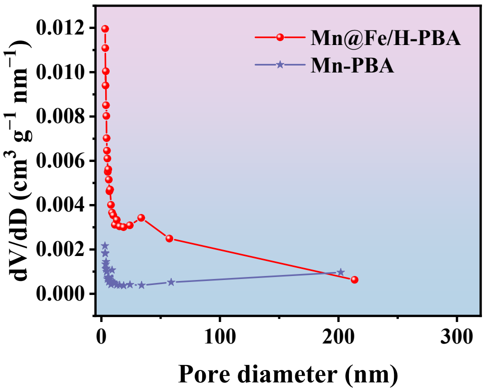

Fig. S8. Pore size distribution curves of Mn@Fe/H-PBA.

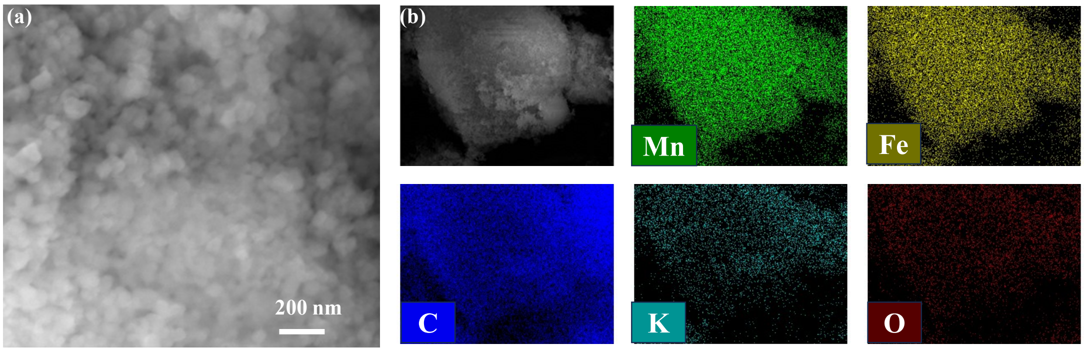

Fig. S9. (a, b) SEM mapping images of Mn−PBA.

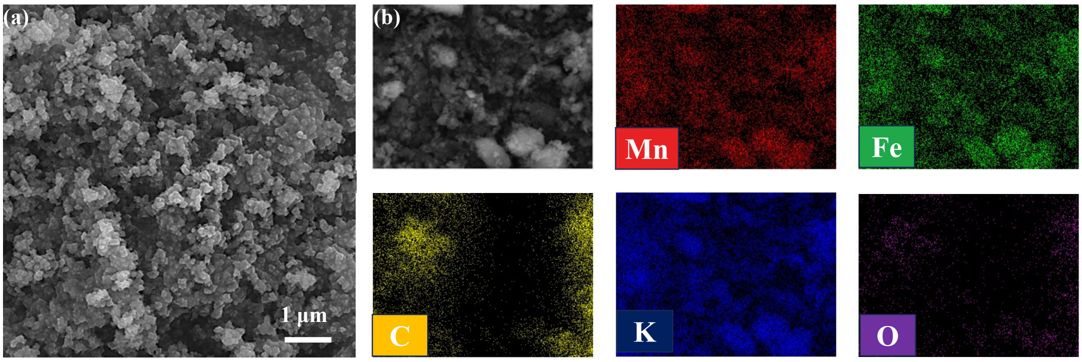

Fig. S10. (a, b) SEM and elemental mapping images of Mn@Fe/H-PBA.

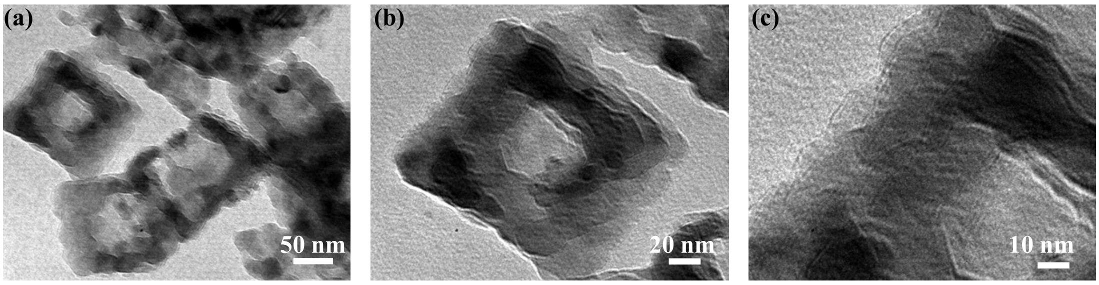

Fig. S11. (a-c) TEM images of Mn@Fe/H-PBA.

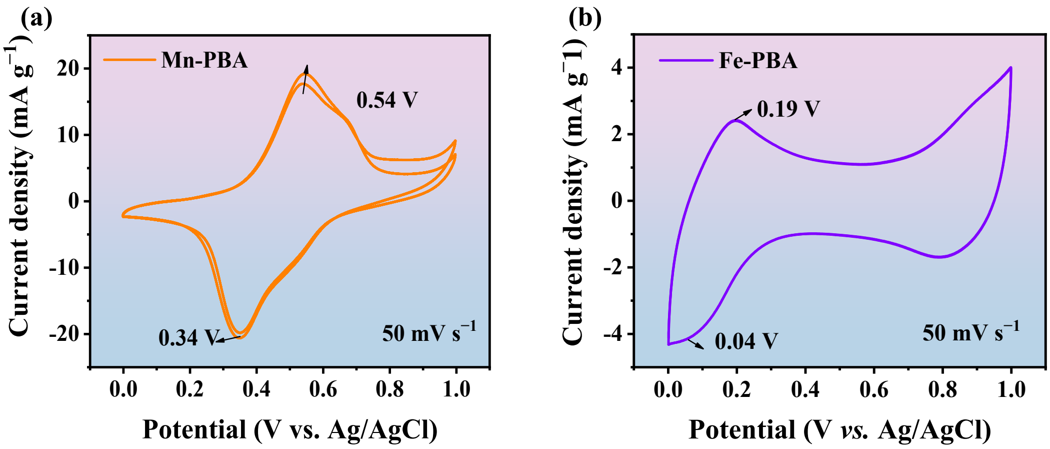

Fig. S12. The CV curves at 50 mV s^{-1} of (a) Mn-PBA and (b)Fe-PBA.

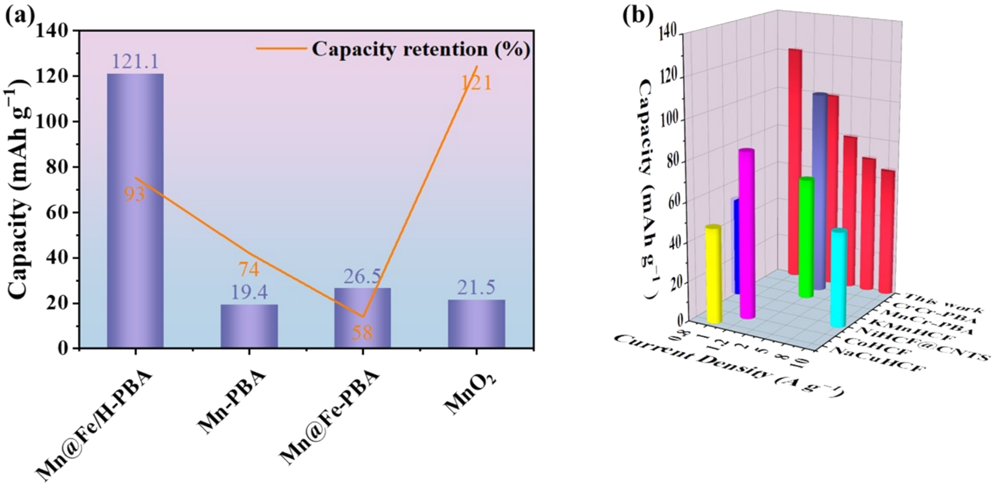

Fig. S13. Performance comparisons: (a) Discharge capacity and capacity retention of Mn@Fe/H-PBA, Mn-PBA, Mn@Fe-PBA, and MnO_2; (b) Comparison of Mn@Fe/H-PBA specific capacitance with other materials at varying current densities.

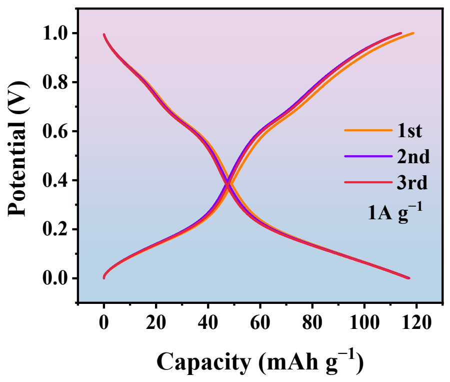

Fig. S14. The GCD curves of Mn@Fe/H-PBA at 1 A g^{-1}

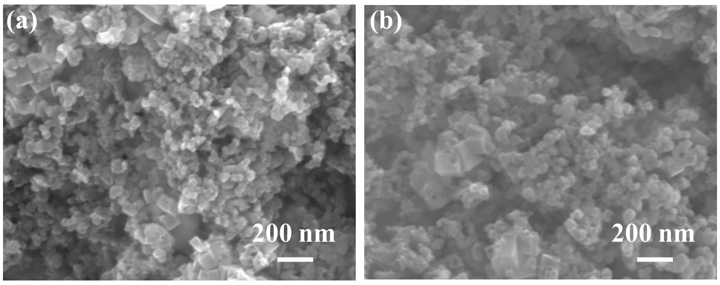

Fig. S15. SEM images of the Mn@Fe/H-PBA electrode (a) before and (b)after cycling.

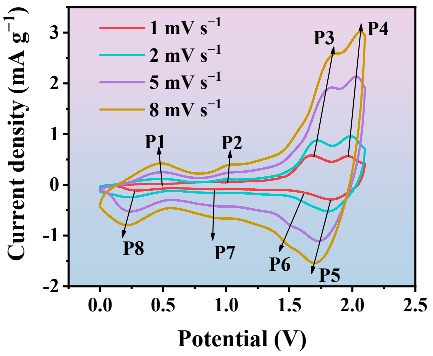

Fig. S16. CV curves at 1-8 mV s^{-1} of Mn@Fe/H-PBA//PI//Na full cell.

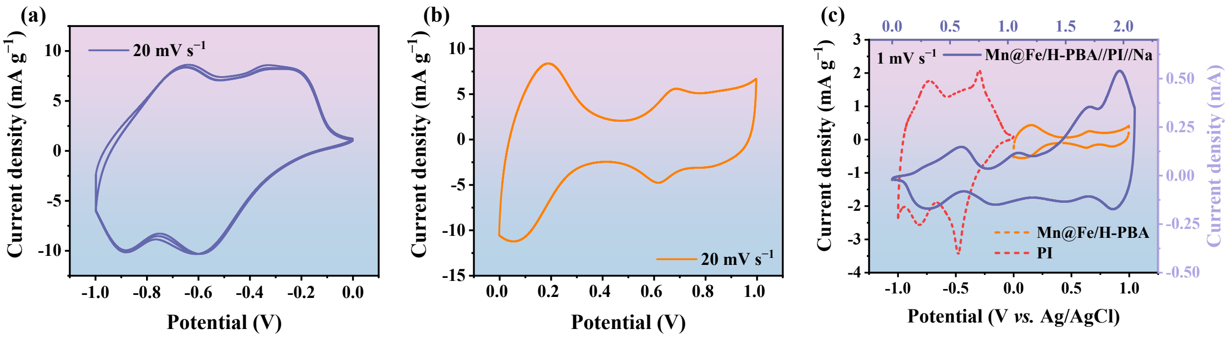

Fig. S17. The first three CV curves at 20 mV s^{-1} of (a) PI, (b) Mn@Fe/H-PBA. (c) CV curves at 1 mV s^{-1}.

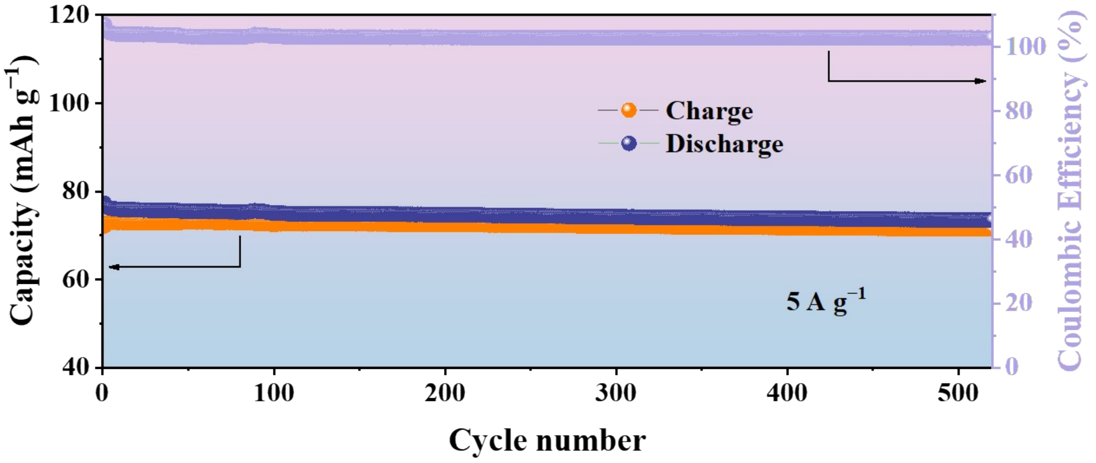

Fig. S18. Cycling performance of PI at 5 A g^{-1}.

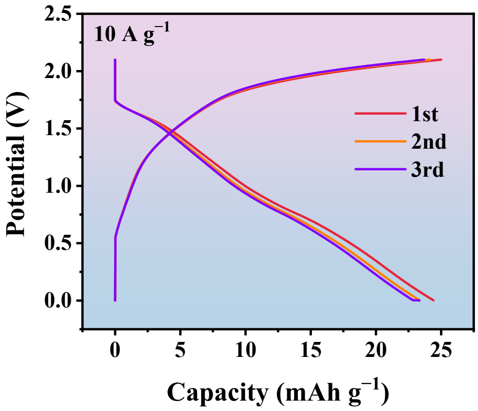

Fig. S19. The GCD curves of Mn@Fe/H-PBA//PI//Na at 10 A g^{-1}.

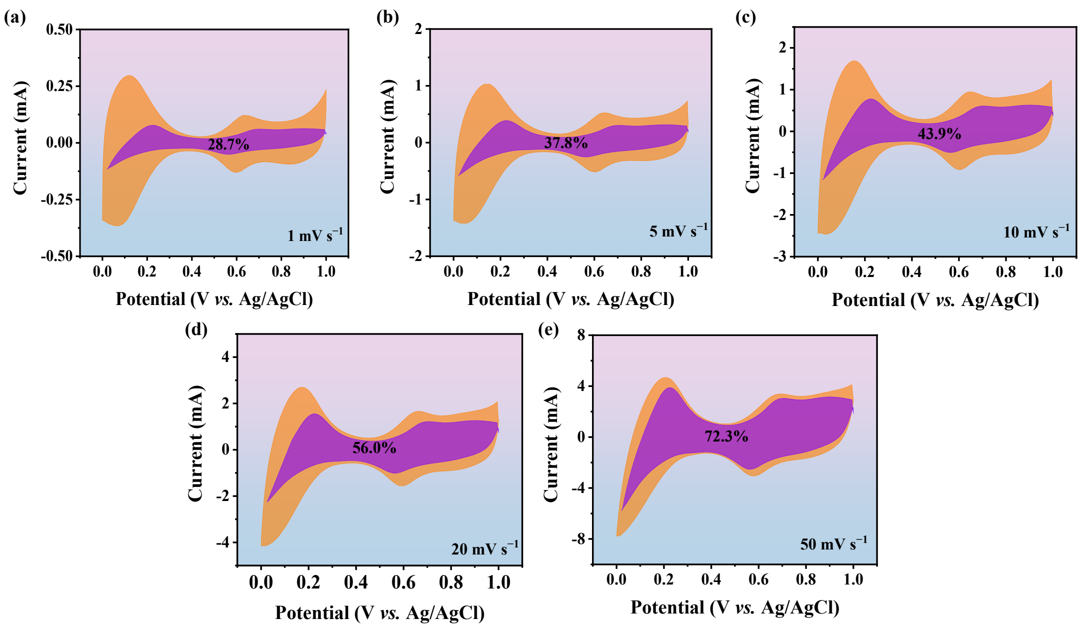

Fig. S20. Capacitance contribution rate at different sweep speeds, (a) 1 mV s^{-1}, (b) 5 mV s^{-1}, (c) 10 mV s^{-1}, (d) 20 mV s^{-1}, and (e) 50 mV s^{-1}.

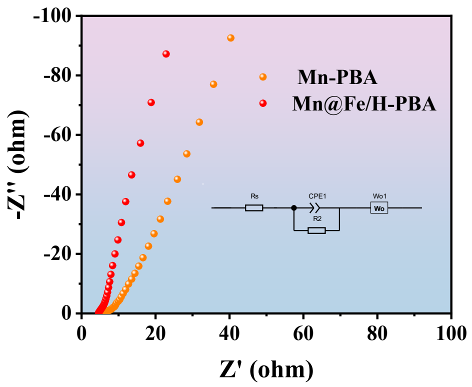

Fig. S21. Nyquist plots (inset shows the equivalent circuit)

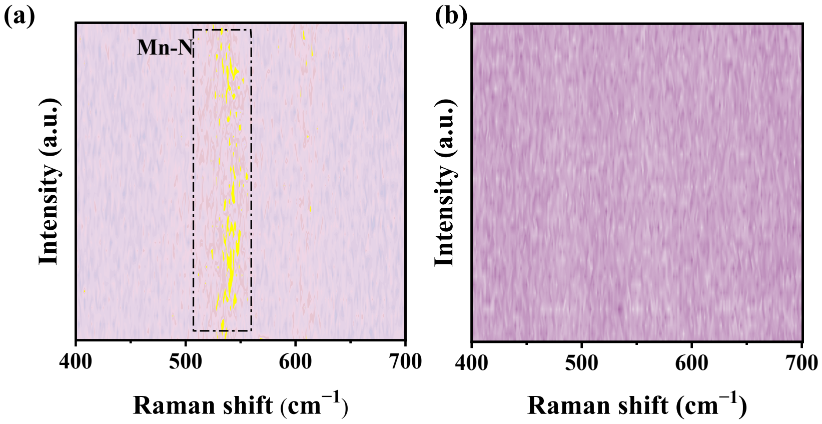

Fig. S22. In-situ Raman spectra analysis of Mn-N: (a) Mn@Fe/H-PBA and (b) Mn-PBA.

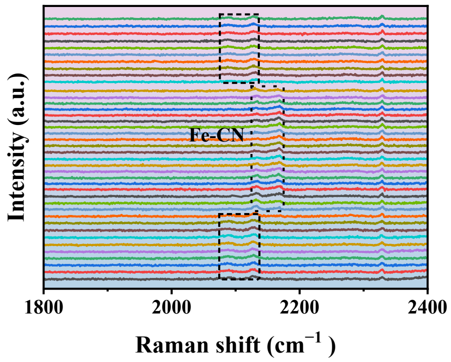

Fig. S23. In-situ Raman plots and spectral analysis of Mn-PBA.

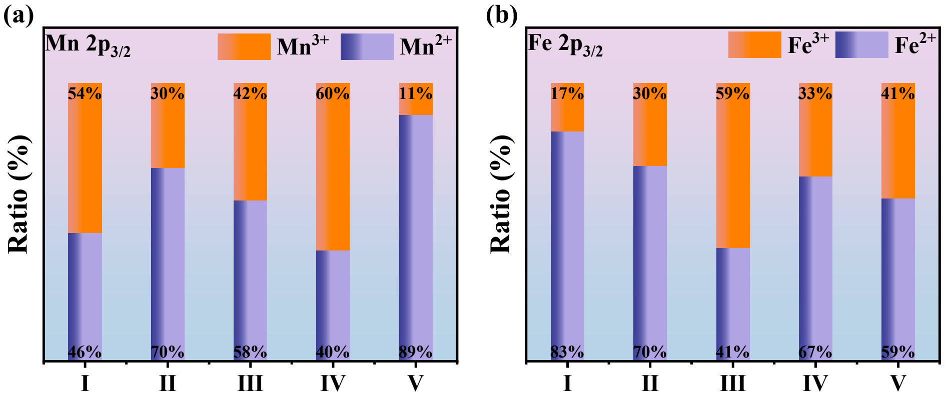

Fig. S24. Changes in the valence of elements, (a) Mn $2p_{3/2}$ and (b) Fe $2p_{3/2}$ in the electrochemical process,

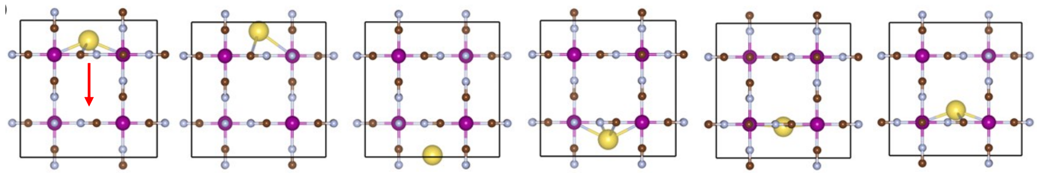

Fig. S25. Diffusion path for Na ion in Mn-PBA

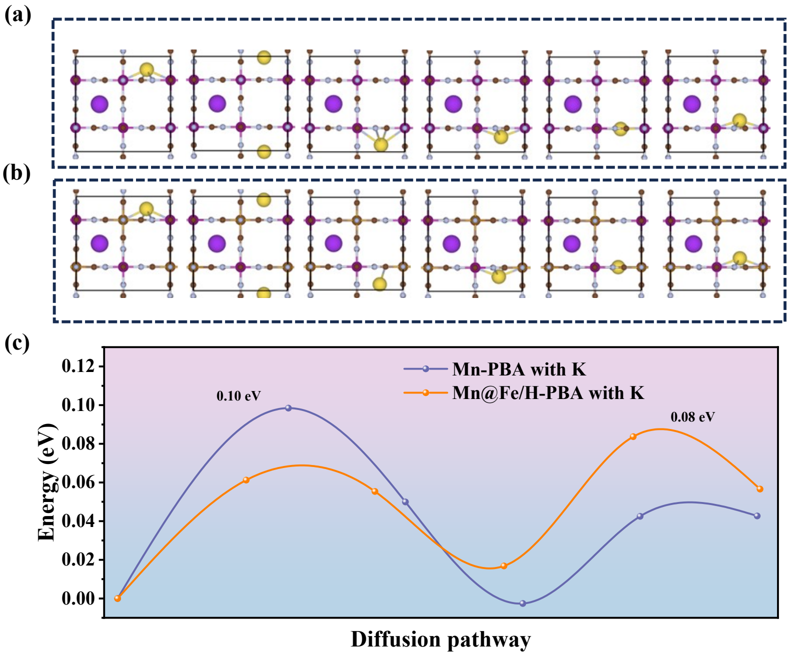

Fig. S26. Diffusion pathway of Na^+ in (a) Mn-PBA and (b) Mn@Fe/H-PBA in the presence of K^+, (c) comparison of Na^+ diffusion energy barriers in Mn-PBA and Mn@Fe/H-PBA.

Table. S1. Electrochemical performances of ASIBs using Mn@Fe/H-PBA in comparison with other PBAs.

Material	Current Density (A g^{-1})	Capacity (mAh A g^{-1})	Refs.
NaCuHCF	1	48	[1]
CoHCF	1.2	84	[2]
NiHCF@CNTs	10	47.9	[3]
KMnHCF	0.8	50	[4]
CrCr-PBA	2	102.5	[5]
This work	1, 2, 5, 8, 10	121, 99.6, 79.5, 69.7, 65	This work

Table. S2. Comparison of the existing full battery performances with this work.

Systems	Energy density (Wh Kg^{-1})	Average Voltage (V)	Ref.
MnPBA@NiHCF//PI	49.9	1.2	[6]
NaCuHCF//NTP	47	1.4	[7]
$NaFePO_4$//NTP	30	0.6	[8]
CuHCFe//MnHCMn	27	0.95	[9]
$Na_4Mn_9O_{18}$//AC	45	0.8	[10]
FeHCF//CuHCF	50	0.7	[11]
$Na_{0.44}MnO_2$//NTP	33	1.1	[12]
$Na_2Mn[Fe(CN)_6]$//$KM[Cr(CN)_6]$	23	1.65	[13]
PPy@MoO_3//$Na_{0.35}MnO_2$	24	1	[14]
Ni_2ZnHCF//PTCDI	40	0.7	[15]
Mn@Fe/H-PBA//PI	74.32	1.15	This work

Table. S3. Comparison of the Rs and Rct of Mn@Fe/H-PBA and Mn-PBA.

Matertial	Rs (Ohm.cm^2)	Rct (Ohm.cm^2)
Mn@Fe/H-PBA	4.97	0.95
Mn-PBA	4.99	1.17

Table S4. The corresponding range of the Na^+ diffusion coefficients of Mn@Fe/H-PBA at different potentials

Potential (V)	Rs (Ohm.cm^2)	Rct (Ohm.cm^2)	D_{Na^+} (cm^2 s^{-1} 10^{10})
0	4.97	0.95	10.23
0.025	4.86	0.97	7.19
0.143	4.87	1.1	4.85
0.300	4.83	1.19	3.65
0.467	4.73	1.25	3.27
0.650	4.67	1.29	2.71
0.810	4.59	1.26	2.02
1.000	4.56	1.22	1.83
0.937	4.52	1.17	1.51
0.753	4.78	1.20	1.65
0.620	4.63	1.28	2.46
0.511	4.69	1.28	2.94
0.300	4.84	1.12	4.67
0.078	4.88	1.08	6.45
0	4.9	0.88	8.52

References

[1] T.-Y. Pan, Ruqia, C.-Y. Wu, C.-S. Ni, S. Gull, A. Haider, H.-Y. Chen, Improvement in cycling stability of Prussian blue analog-based aqueous sodium-ion batteries by ligand substitution and electrolyte optimization, *Electrochim. Acta*, 427 (2022) 140778.

[2] T. Shao, C. Li, C. Liu, W. Deng, W. Wang, M. Xue, R. Li, Electrolyte regulation enhances the stability of Prussian blue analogues in aqueous Na-ion storage, *J. Mater. Chem. A*, 7 (2019) 1749-1755.

[3] Y. Yuan, D. Bin, X. Dong, Y. Wang, C. Wang, Y. Xia, Intercalation Pseudocapacitive Nanoscale Nickel Hexacyanoferrate@Carbon Nanotubes as a High-Rate Cathode Material for Aqueous Sodium-Ion Battery, *ACS Sustainable Chem. Eng.*, 8 (2020) 3655-3663.

[4] J. Liu, C. Yang, B. Wen, B. Li, Y. Liu, Ultra-Long Cycle of Prussian Blue Analogs Achieved by Equilibrium Electrolyte for Aqueous Sodium-Ion Batteries, *Small*, 19 (2023) 2303896.

[5] J. Chen, C. Liu, Z. Yu, J. Qu, C. Wang, L. Lai, L. Wei, Y. Chen, High-energy-density aqueous sodium-ion batteries enabled by chromium hexacycnochromate anodes, *Chem. Eng. J.*, 415 (2021) 129003.

[6] L. Ye, H. Fu, J. Ji, Z. Wu, H. Ren, Y. Zhang, M. Shi, J. Yang, Bilayer Mn-based Prussian blue cathode with high redox activity for boosting stable cycling in aqueous sodium-ion half/full batteries, *J. Colloid Interface Sci.*, 684 (2025) 635-646.

[7] X.Y. Wu, M.Y. Sun, Y.F. Shen, J.F. Qian, Y.L. Cao, X.P. Ai, H.X. Yang, Energetic Aqueous Rechargeable Sodium-Ion Battery Based on $Na_2CuFe(CN)_6$-$NaTi_2(PO_4)_3$ Intercalation Chemistry, *Chemsuschem*, 7 (2014) 407-411.

[8] A.J. Fernández-Ropero, D. Saurel, B. Acebedo, T. Rojo, M. Casas-Cabanas, Electrochemical characterization of $NaFePO_4$ as positive electrode in aqueous sodium-ion batteries, *J. Power Sources*, 291 (2015) 40-45.

[9] M. Pasta, C.D. Wessells, N. Liu, J. Nelson, M.T. McDowell, R.A. Huggins, M.F. Toney, Y. Cui, Full open-framework batteries for stationary energy storage, *Nat. Commun.*, 5 (2014) 3007.

[10] J.F. Whitacre, A. Tevar, S. Sharma, $Na_4Mn_9O_{18}$ as a positive electrode material for an aqueous electrolyte sodium-ion energy storage device, *Electrochem. Commun.*, 12 (2010) 463-466.

[11] B.Q. Wang, X. Wang, C. Liang, M. Yan, Y.Z. Jiang, An All-Prussian-Blue-Based Aqueous Sodium-Ion Battery, *Chemelectrochem*, 6 (2019) 4848-4853.

[12] Z. Li, D. Young, K. Xiang, W.C. Carter, Y.M. Chiang, Towards High Power High Energy Aqueous Sodium-Ion Batteries: The $NaTi_2(PO_4)_3/Na_{0.44}MnO_2$ System, *Adv. Energy Mater.*, 3 (2013) 290-294.

[13] K. Nakamoto, R. Sakamoto, Y. Sawada, M. Ito, S. Okada, Over 2 V Aqueous Sodium-Ion Battery with Prussian Blue-Type Electrodes, *Small Methods*, 3 (2019) 1800220.

[14] Y. Liu, B.H. Zhang, S.Y. Xiao, L.L. Liu, Z.B. Wen, Y.P. Wu, A nanocomposite of MoO_3 coated with PPy as an anode material for aqueous sodium rechargeable batteries with excellent electrochemical performance, *Electrochim. Acta*, 116 (2014) 512-517.

[15] Y.F. Sun, Y. Zhang, Z. Xu, W.S. Gou, X.G. Han, M.M. Liu, C.M. Li, Dilute Hybrid Electrolyte for Low-Temperature Aqueous Sodium-Ion Batteries, *Chemsuschem*, 15 (2022) e202201362.
